# Quantitative and sensitive RNA based detection of *Bacillus* spores

**DOI:** 10.3389/fmicb.2014.00092

**Published:** 2014-03-11

**Authors:** Ekaterina Osmekhina, Antonina Shvetsova, Maria Ruottinen, Peter Neubauer

**Affiliations:** ^1^Department of Process and Environmental Engineering and Biocenter Oulu, University of OuluOulu, Finland; ^2^Department of Biochemistry and Biocenter Oulu, University of OuluOulu, Finland; ^3^Laboratory of Bioprocess Engineering, Department of Biotechnology, Technische Universität BerlinBerlin, Germany

**Keywords:** spore detection, *Bacillus subtilis*, RNA hybridization

## Abstract

The fast and reliable detection of bacterial spores is of great importance and still remains a challenge. Here we describe a direct RNA-based diagnostic method for the specific detection of viable bacterial spores which does not depends on an enzymatic amplification step and therefore is directly appropriate for quantification. The procedure includes the following steps: (i) heat activation of spores, (ii) germination and enrichment cultivation, (iii) cell lysis, and (iv) analysis of 16S rRNA in crude cell lysates using a sandwich hybridization assay. The sensitivity of the method is dependent on the cultivation time and the detection limit; it is possible to detect 10 spores per ml when the RNA analysis is performed after 6 h of enrichment cultivation. At spore concentrations above 10^6^ spores per ml the cultivation time can be shortened to 30 min. Total analysis times are in the range of 2–8 h depending on the spore concentration in samples. The developed procedure is optimized at the example of *Bacillus subtilis* spores but should be applicable to other organisms. The new method can easily be modified for other target RNAs and is suitable for specific detection of spores from known groups of organisms.

## Introduction

Detection of microorganisms in air is challenging due to the very low amount of organisms that generally are collected in air samples and the presence of organisms as dormant spores.

Various biosensors have been developed for the specific detection of harmful microbial spores in air samples (Gooding, 2006). Most methods for the detection of *Bacillus* spores have been designed for *Bacillus anthracis*, a causative factor for anthrax and a potential biological threat agent (Edwards et al., 2006; Irenge and Gala, 2012), in order to create sensors for biological warfare agents. Being a causative agent, *B. anthracis* is difficult to work with, and therefore the closely related species *Bacillus subtilis* and *B. cereus* are often used in the development of detection strategies (Arakawa et al., 2003; Stachowiak et al., 2007; Inami et al., 2009; Cheng et al., 2011).

The primary strategies for detection of *Bacillus* spores include polymerase chain reaction based techniques, immunoassays, spectrometry, chromatography, and protein profiling (Table 1). Recently also some autonomous pathogen detection systems for aerosol collection, sample preparation and detection were developed (Hindson et al., 2005; Stachowiak et al., 2007; Regan et al., 2008; Inami et al., 2009) (Table 1).

**Table 1:** **Methods for detection of *Bacillus* spores**.

**Method**	**Sensitivity**	**Organisms**	**Comments**	**References**
**NUCLEIC ACID BASED METHODS**				
Real-time PCR	1 spore per 100 L of air	*B. anthracis*		Lee et al., 1999; Makino and Cheun, 2003; Irenge et al., 2010; Wielinga et al., 2011
Culture-based PCR	1–10 spores per analysis	*B. anthracis*	High-throughput	Kane et al., 2009
NASBA^a^ coupled with biosensor	1–10 spores per analysis	*B. anthracis*	Analysis after 30 min of germination	Baeumner et al., 2004
FISH^b^	10^3^ spores per m^3^ of air	*B. anthracis*		Weerasekara et al., 2013
ICAN^c^ DNA detection	10^4^ spores per analysis	*B. subtilis*	Does not contain a germination step	Inami et al., 2009
Autonomous pathogen detection system with multiplexed PCR		*B. anthracis* and others		Regan et al., 2008
RAZOR^®^ EX Anthrax Air Detection System	200 spores per analysis	*B. anthracis*	DNA extraction and real-time PCR	Spaulding et al., 2012; Hadfield et al., 2013
**IMMUNOASSAYS**				
Colorimetric and electrochemilumine-scence immunoassay	30–100 spores per analysis	*B. anthracis*		Morel et al., 2012
ELISA^d^		*B. subtilis*		Zhou et al., 2002
On-chip ELISA	10^5^ spores per analysis	*B. subtilis*	Chemiluminescence method combined with a biochip. Antibodies against surface spore antigen were used	Stratis-Cullum et al., 2003
Luminex assay	10^3^–10^4^ spores per ml	*B. anthracis*	Monoclonal antibodies recog-nize anthrose-containing oligosaccharides on the surface of *B. anthracis* endospores	Tamborrini et al., 2010
Peptide-Function cantilever arrays	10^5^ spores per ml for analysis	*B. subtilis*	1 from 2400 spores was captured	Dhayal et al., 2006; Campbell and Mutharasan, 2007
Chip gel electrophoresis protein profiling (CGE-PP)	16 particles per liter 100 cells per analysis	Any (adapted for *E. coli* and *B. subtilis*)	Autonomous microfluidic system	Pizarro et al., 2007; Stachowiak et al., 2007
Multiplexed Immunoassay with PCR Confirmation	49 spores per liter of air	*B. subtilis*	Autonomous Detection of Aerosolized Biological Agents	McBride et al., 2003; Hindson et al., 2004
**OTHERS**				
Pyrolysis micromachined differential mobility spectrometry	10^3^ spores per analysis	*B. anthracis*	A microfabricated ion mobility spectrometer in combination with a pattern recognition and classification algorithm	Krebs et al., 2006
Microcalorimetric spectroscopy	100–1000 spores	*B. subtilis, B. cereus*		Arakawa et al., 2003
Laser induced breakdown spectroscopy	single particles	*B. subtilis*	In combination with other detection methods	Hybl et al., 2003, 2006
Mass-spectrometry	10^4^–10^5^ spores	*B. anthracis*	In combination with other detection methods	Lasch et al., 2009; Chenau et al., 2011; Li et al., 2012
Raman scattering	10^4^ spores	Not specific	Based on detection of dipicolinic acid	Cheng et al., 2011, 2012; Cowcher et al., 2013
Optical microchip array biosensor	5 × 10^7^ spores per ml	*B. anthracis* and others		Bhatta et al., 2011

a NASBA, Nucleic acid sequence based amplification.

b FISH, Fluorescence in situ hybridization.

c ICAN, Isothermal and chimeric primer-initiated amplification of nucleic acids.

d ELISA, Enzyme linked immunosorbent assay.

A number of direct methods are based on DNA or protein detection and cannot distinguish between viable and dead spores. Furthermore, most of them have low sensitivity or require an amplification step such as PCR. RNA-based detection methods have the advantage to specifically analyze only viable spores, and RNA's, especially ribosomal RNAs (rRNAs), are populated in high amounts, making enzyme-based amplifications methods indispensible. Thus it is reasonable to apply RNA detection after the activation of RNA synthesis which takes place already after approximately 10 min of germination (Keijser et al., 2007).

Sandwich hybridization assays (SHA) are suitable for a rapid and quantitative RNA detection (Rautio et al., 2003). These methods are based on the hybridization of a target RNA (or denatured DNA) with two specific oligonucleotide probes. A capture probe is used to immobilize the target on a solid support, such as magnetic microbeads, which provide a large surface area for nucleic acid attachments (Walsh et al., 2001). This binding between the probes and the beads is usually performed by interaction between biotin attached to the oligonucleotide probe and streptavidin coated magnetic beads. The detection probe is labeled with a marker molecule which generates a signal proportional to the amount of target molecules. Oligonucleotide probes required for this assay can be designed for almost any RNA and can easily be modified for other targets. This means that the developed detection system can be applied for different organisms with just some small adaptations. Sandwich hybridization is relatively sensitive (10^−16^–10^−15^ moles of a specific target molecule) and can be performed with crude biological samples without any RNA purification. The method has been successfully applied for the detection of 16S rRNA from *Legionella* sp. in water samples (Leskelä et al., 2005), mycobacteria in soils (Nieminen et al., 2006), *Lactobacillus* and *Pediococcus* in brewery yeast slurries (Huhtamella et al., 2007), *Salmonella* in minced meat (Taskila et al., 2011), *Bacillus cereus* DNA (Gabig-Ciminska et al., 2004) and for monitoring dynamic changes of different mRNA species in microbial processes (Rautio et al., 2003; Neubauer et al., 2007; Soini et al., 2008; Thieme et al., 2008). The possibility to use various markers makes the method applicable for different read-out systems, such as fluorescence meters (Rautio et al., 2003), chip-based fluorescent biosensors (Wang et al., 2013) or electrical biochip readers (Gabig-Ciminska et al., 2004; Jürgen et al., 2005; Elsholz et al., 2006; Pioch et al., 2008a,b). The SHA coupled with capillary electrophoresis called TRAC (transcript analysis with aid of affinity capture) was developed for multiplex transcript analysis and is commercially available (Rautio et al., 2006, 2008; Rautio, 2010) (PlexPress Oy, Finland).

In the actual study a procedure was developed for detecting bacterial spores, utilizing spore activation, enrichment cultivation and an RNA-based sandwich hybridization assay. In contrast to most assays utilizing DNA and protein detection, the method developed here is specific only for viable organisms since it is based on RNA synthesis.

## Materials and methods

### Spore preparation

*Bacillus subtilis* spores were obtained from cells (*B. subtilis* 6051α, kindly provided by Prof. Dr. Thomas Schweder, Ernst-Moritz-Arndt University of Greifswald, Germany) cultured in Schaeffer's sporulation medium (8 g/l bacto-nutrient broth, 0.1% (w/v) KCl, 0.012% (w/v) MgSO_4_ × 7H_2_0, 0.5 mM NaOH, 1 mM Ca(NO_3_)_2_, 0.01 mM MnCl_2_, 0.001 mM FeSO_4_) at 37°C, 200 rpm for 4 days. Cultures were carried out in 3-baffled 1 liter Erlenmeyer flasks with a liquid volume of 100 ml. After harvesting and extensive washing with water at 4°C, spores were inspected by phase-contrast microscopy to show that samples are free of vegetative cells (< 10%). Spores were lyophilized and stored at 4°C. For counting, the spores were diluted in 0.9% NaCl, activated at 70°C for 30 min and plated on nutrient agar plates (Difco, USA).

### Activation and growth conditions

*B. subtilis* spores were diluted in 0.9% NaCl and activated by temperature treatment at 70°C for 30 min. Thereafter the suspensions were transferred into 3-baffled 1 l Erlenmeyer flasks containing 100 ml of a germination medium (20 g/l tryptone, 10 g/l yeast extract, 10 g/l NaCl and 0.5 mM L-alanine) (Moeller et al., 2006). Cultures were carried out at 37°C and 200 rpm on a rotary shaker. Culture growth was monitored by measuring the optical density at 600 nm (OD_600_, Ultrospec Pro 2100 UV/Visible Spectrophotometer, GE Healthcare, Buckinghamshire, UK). The corresponding colony forming units (cfu/ml) were determined by plating on nutrient agar plates.

### Sample preparation

1 ml of the *B. subtilis* cultures were removed into pre-cooled 1.5 ml microreaction tubes containing 0.125 × sample volume of cold 95:5 ethanol:phenol (v/v) (Thieme et al., 2008). The cells were collected by centrifugation (15,000 × g, + 4°C, 5 min). The pellets were suspended in 500 μl of 1 × TEN buffer (10 mM Tris-HCl, 1 mM EDTA, 100 mM NaCl, pH 8.0) containing 1 μl/ml RNAguard^®^ RNase inhibitor (Amersham Biosciences, New Jersey, USA) and mixed by vortexing for 1 min. Then the cell suspensions were transferred into 2 ml microcentrifuge tubes with a skirt (Greiner Bio-One GmbH, Frickenhausen, Germany) containing 100 mg glass beads (BioSpec Products Inc., Bartlesville, OK, USA) with a diameter of 0.1 mm. Cell disruption was performed with a FastPrep^®^ FP120 Cell Disrupter (Bio-101, Thermo Savant, USA) at 4.5 m/s for 4 × 25 s. The vials were kept on ice for 1 min between the disruption cycles. Cell homogenates were centrifuged (20,000 ×g, + 4°C, 5 min) and supernatants were snap frozen in liquid nitrogen.

### Generation of the DNA for the *in vitro* 16S rRNA transcript

The target gene was amplified by PCR using a colony of *B. subtilis* cells as a template and 10 pmol of the forward and reverse primers (Weisburg et al., 1991) (Table 2). The forward primer contained at the 5′–end the T7 promoter sequence CTA ATA CGA CTC ACT ATA GGG. The PCR conditions were as follows: 3 min at 95°C; 35 cycles of 15 s at 95°C, 30 s at 50°C and 1 min 45 s at 72°C; 10 min at 72°C and afterwards held at 4°C. PCR products were analyzed by agarose gel electrophoresis and purified with the QIAquick PCR purification kit (Qiagen, Hilden, Germany). Purified PCR products were quantified by absorbance measurement at 260 nm (GeneQuant II, Viochrom Ltd., Cambridge, UK).

**Table 2:** **Sequences of the oligonucleotide probes used in sandwich hybridization for the detection of *B. subtilis* 16S rRNA and PCR primers**.

**Probe name**	**Probe specification**	**Probe sequence 5′–3′**	**Location of probe**	**Probe modification**
Bsub16Scap	Capture	TGTCTCAGT**C**CCAGTGTGG^a^	319–337	5′-Biotin
Bsub16Sdet	Detection	CGTAGGAGTCTGG**G**CCG^a^	338–354	3′-Digoxigenin
Help1	Helper	CCCCACTGCTGCCTCC	355–370	
Help2	Helper	CTGGTCATCCTCTCAGA	302–318	
Fd-T7	Forward PCR primer	(CTAATACGACTCACTATAGGG) AGAGTTTGATCCTGGCTCAG	10–29	5′-T7 promoter
Rev	Reverse PCR primer	CGGCTACCTTGTTACGACTT	1502–1521	
NCcap	Negative control capture probe	TGTGAACTTCCATCGGCTTGAGCC		5′-Biotin
NCdet	Negative control detection probe	GATAGTCCCTCTAAGAAGCCATGTG		3′-Digoxigenin

a Nucleotides different to general eubacterial probes are underlined.

Target RNA was generated from the purified PCR product by means of T7 RNA polymerase *in vitro* transcription using the MAXIscript™ T7-kit (Ambion, Austin, TX, USA). The quality of the *in vitro* transcribed RNA was checked by the Agilent 2100 Bioanalyser and RNA 6000 Nano LabChip Kit (Agilent Technologies, Waldbronn, Germany). RNA was quantified by absorbance measurement at 260 nm (NanoDrop ND 1000, Thermo Fisher Scientific, USA).

### Oligonucleotide probes

The oligonucleotide probes for *B. subtilis* 16S rRNA were designed on the basis of general eubacterial probes with some modifications (Table 2). The oligonucleotide probes were purchased from Oligomer Oy (Helsinki, Finland). The detection probe was labeled with the DIG Oligonucleotide Tailing Kit (Roche Diagnostics, Mannheim, Germany) according to the manufacturer's instructions.

### Sandwich hybridization assay

A quantitative sandwich hybridization assay (SHA) was used for detecting 16S rRNA molecules. The procedure was performed as described by Rautio et al. (2003) and Thieme et al. (2008) with small modifications.

SHAs were carried out in 96-well bright U-shaped microplates (Greiner-Bio-One GmbH, Frickenhausen, Germany) using a Thermomixer Comfort (Eppendorf, Hamburg, Germany). The target RNA was mixed with the 5 × SSC hybridization buffer (0.75 M sodium chloride, 0.075 M sodium citrate, pH 7.0), 20% (v/v) deionized formamide, 3% (w/v) dextran sulphate, 0.2% (v/v) TWEEN20, 0.02% (v/v) Ficoll, 0.02% (w/v) polyvinyl pyrrolidone, 0.02% (w/v) bovine serum albumin, 1% (v/v) blocking reagent (Roche, Mannheim, Germany) in 100 mM maleic acid with 150 mM NaCl (pH 7.5), 5 pmol biotin-labeled capture probe, 1 pmol DIG-labeled detection probe, and 1 pmol of each helper probe in a total volume of 100 μl. The hybridization reactions were performed in three parallels at 750 rpm and 50°C for 30 min. 15 μl of Streptavidin MagneSphere^®^ Paramagnetic Particles (Promega, Madison, WI, USA) were washed as recommended by the manufacturer, and added to each well after the hybridization reaction and incubated at 750 rpm and 50°C for 30 min. Subsequently, beads were washed three times with 120 μl of a washing buffer (1% SSC, 0.1% TWEEN20) at 25°C and 750 rpm for 2 min. During the washing step beads were kept in the wells with a MagnaBot^®^ 96 Magnetic Separation Device (Promega, Madison, WI, USA). 100 μl of anti-DIG alkaline phosphatase Fab-fragments (Roche Diagnostics, Mannheim, Germany) were diluted with 1 × SSC, 0.1% TWEEN20 to a final concentration of 375 U/l, and applied to each well. The 96-well plate was incubated at 450 rpm and 25°C for 30 min. Unbound enzyme was removed by washing three times as described above. Afterwards the whole solutions were transferred into new microplates and washed once more. Finally, 100 μl of 1 mM AttoPhos^®^ fluorescent substrate (Promega, Madison, WI, USA) was added to each well and the enzymatic reaction was performed at 37°C and 750 rpm for 20 min. 90 μl of the contents of well were transferred into a Costar black 96-well assay plate (Corning Inc., Corning, NY, USA) for the fluorescence measurement with a Wallac Victor multilabel counter (PerkinElmer Life Sciences, Turku, Finland) at an excitation wavelength of 430 nm and an emission wavelength of 560 nm.

The detection limit was defined as 3 × SD added to the background fluorescence. Detection limits were calculated separately for each SHA and signals above it were considered as positive. Capture and detection probes which are not complementary to any RNA of *B. subtilis* (Table 2, NCcap and NCdet, respectively) were used as a negative control for each sample to estimate the background signal.

## Results

**Figure 1:**
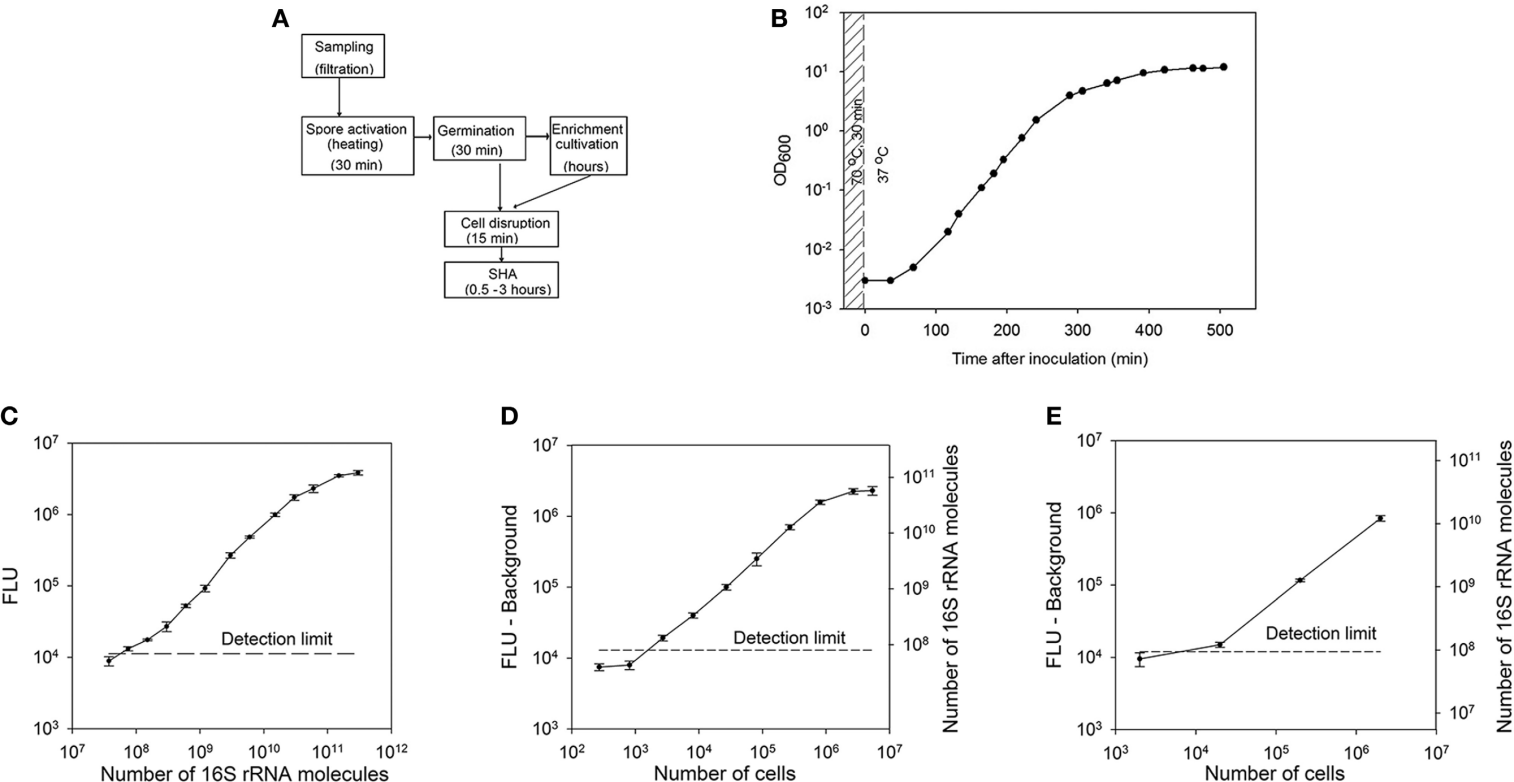
**Spore detection using a sandwich hybridization assay.** (**A**) Principle of airborne spore detection using a sandwich hybridization assay. Spores collected from air are activated and cultivated in a germination medium. The first sample for analysis can be collected after approximately 30 min of germination. If the amount of spores is too low to be detected at this step, the incubation can be continued to allow a multiplication of the cells. (**B**) A Growth curve of *B. subtilis* cells. After heat activation (70°C for 30 min) *B. subtilis* spores were inoculated into a germination medium to a final concentration of 10^6^ spores/ml. (**C**) Standard curve for the *B. subtilis* 16S rRNA sandwich hybridization assay. (**D**) SHA dilution curve for *B. subtilis* 16S rRNA. The dilutions of the crude extracts of *B. subtilis* cells collected at the exponential growth phase (OD_600_ = 1.4) were used as a target. The corresponding numbers of 16S rRNA molecules were calculated according to the standard curve of *in vitro* transcribed 16SrRNA at 260 nm. (**E**) 16S rRNA measured with SHA in *B. subtilis* spores after their activation and germination for 30 min. Corresponding numbers of 16S rRNA molecules were calculated according to the standard curve (Figure 1C). The error bars show the ±*SD* of three parallel experiments and the detection limit is shown as a dashed horizontal line.

The aim of this study was the development of a diagnostic method for the detection of bacterial spores at the example of *Bacillus subtilis* spores. The sequence of the procedure is shown in Figure 1A. First, the spores are activated by a heat treatment (30 min, 70°C) and following transfer into germination medium (Keijser et al., 2007). The duration of the enrichment cultivation was varied depending on the initial amount of spores. After the incubation phase collected samples were disrupted and the supernatant was utilized for the quantification of the 16S ribosomal RNA directly from the cell broth by the use of a sandwich hybridization assay without any further purification.

RNA-based detection of spores is feasible only after the activation of RNA synthesis, that takes place during the spore germination. In order to activate *B. subtilis* spores and initiate the germination process, dormant spores were exposed to heat (70°C for 30 min) and afterwards cultured in the germination medium at 37°C. Cell germination and growth were monitored by measuring the OD_600_ (Figure 1B). An increase of the cell density after 1 h of cultivation indicated that the germination and outgrowth were completed and proliferation began.

For this feasibility study oligonucleotide probes for the detection of *B. subtilis* 16S rRNA with SHA were designed on the basis of general eubacterial probes with some modifications to detect the sequences specific for *B. subtilis* (Table 2). The probes were tested using an *in vitro* transcribed fragment of *B. subtilis* 16S rRNA as target molecule, which also is applied as a quantitative standard. 6 × 10^7^ (0.1 fmol) target molecules in the hybridization solution gave a signal that was significantly above the detection limit. The reaction was linear over a range of nearly 3 orders of magnitude up to approximately 3 × 10^10^ i.e., 50 fmol of target molecules (Figure 1C).

In order to determine the detection limit and the range of the SHA for crude cell extracts, different dilutions of *B. subtilis* cell extracts were analyzed with the sandwich hybridization assay. The cells were collected at the end of the exponential growth phase (OD_600_ = 1.4), disrupted, and 2.7 × 10^2^ to 5.4 × 10^6^ cells were used as targets for a dilution curve (Figure 1D). The detection limit was about 1.5 × 10^3^ cells per assay for exponentially growing *B. subtilis* cells (7.5 × 10^4^ cells per ml of culture medium). The calculated amount of 16S rRNA was 4.8 (±0.6) × 10^4^ molecules per cell at this growth phase. Consequently, the detection limit in crude cell extracts corresponded to about 7.2 × 10^7^ 16S rRNA molecules. The linear range of the assay was between 10^3^ and 10^6^ cells in a hybridization solution.

In order to establish the analysis of cells from enrichment cultures the germination medium was inoculated with 10^5^–10^8^ activated *B. subtilis* spores per mL. After 30 min of cultivation (before the first proliferation), the cells were disrupted and analyzed with SHA (Figure 1E). It was possible to detect about 20,000 cells per assay (10^6^ cells per ml of culture medium). This number of cells corresponds to approximately 10^8^ 16S rRNA molecules. According to the measurements at this stage of germination one *B. subtilis* cell contained 5.6 (±0.9) × 10^3^ 16S rRNA molecules.

**Figure 2:**
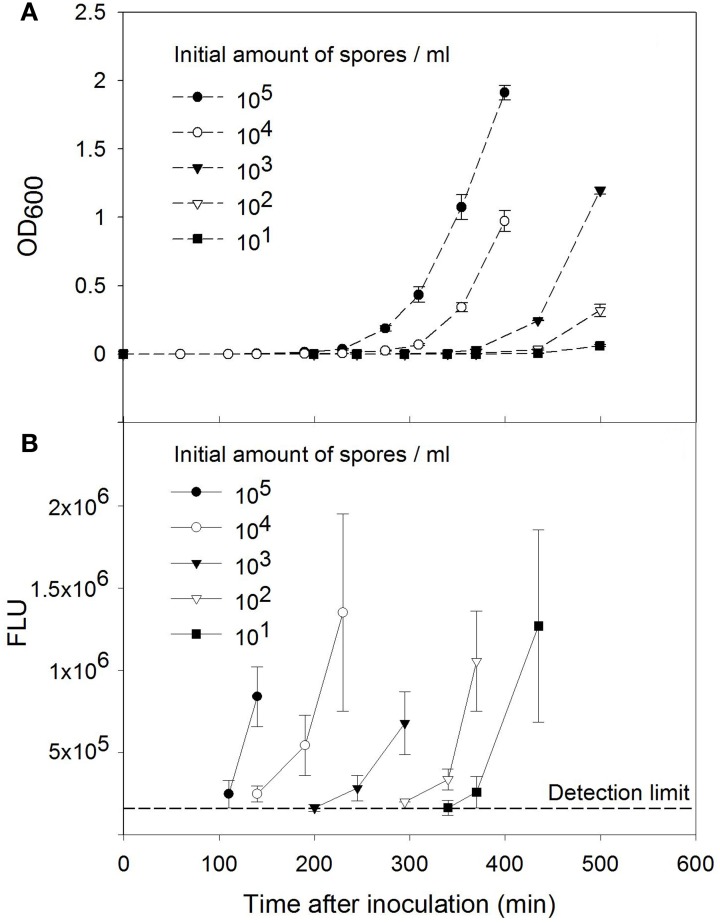
**Sensitivity of the *B. subtilis* spore detection method. (A)** Growth curves of *B. subtilis* cells after spore activation. The initial number of the spores was 10^1^–10^5^ spores per ml of germination medium. **(B)** Detection of *B. subtilis* 16S rRNA by sandwich hybridization after enrichment cultivation. The error bars show ±*SD* of three independent cultivations and measurements.

Next, the sensitivity of the method including activation, enrichment cultivation, and 16S rRNA analysis of *B. subtilis* spores was studied. Therefore the germination medium was inoculated with 10^1^–10^5^ activated *B. subtilis* spores per ml. Germination and growth of the cells were monitored by measuring the OD_600_ (Figure 2A). The cells were collected, disrupted and analyzed with SHA using the probes against the16S rRNA (Figure 2B). Detectable signals were observed after 110, 140, 245, 340, and 370 min of cultivation for the samples with initial number of 10^5^, 10^4^, 10^3^, 10^2^, and 10^1^ spores per ml of culture medium, respectively.

**Figure 3:**
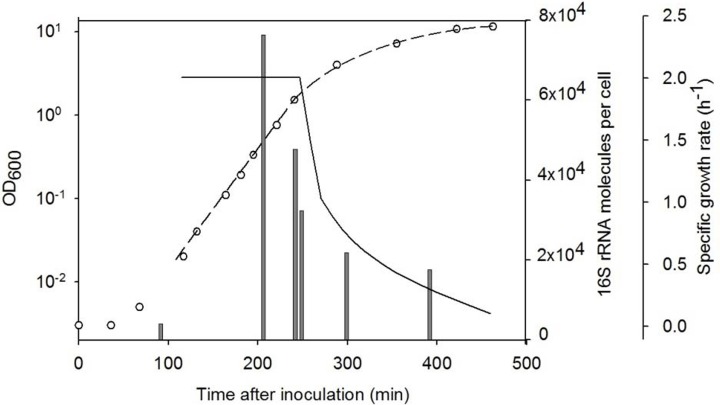
**Level of 16S rRNA molecules per cell (gray bars) in different growth phases of *B. subtilis*.** Quantity of RNA was measured with SHA. The growth was followed by measuring the optical density at 600 nm (◦) and the specific growth rate was calculated (black line). The dashed line shows fitting of the OD curve.

Since the SHA sensitivity depends on the number of target RNA molecules in one cell, the amount of 16S rRNA was measured for different growth phases of *B. subtilis* cells (Figure 3). As it was shown earlier one *B. subtilis* cell contained 5.6 (±0.9) × 10^3^ 16S rRNA molecules already after 30 min of germination. At the early exponential growth phase the amount of 16S rRNA molecules per cell was maximal (about 9 × 10^4^ rRNA molecules per cell for an OD_600_ of 0.27) and the 16S rRNA content decreased in later growth stages. Therefore the analysis is most sensitive if the OD_600_ is approximately 0.3.

## Discussion

Detection of microorganisms in bioaerosols is an important issue in determining bio-warfare agents during biological attacks and for the monitoring of indoor air quality. A fast and sensitive method for the detection of bacterial spores was developed in this study. The procedure includes activation of spores, their germination, enrichment cultivation and RNA detection using a sandwich hybridization assay, thus only viable spores are detected, which is advantageous over standard detection methods as listed in Table 1. There are no sensitivity limitations as the method is adaptable by the enrichment cultivation time. The RNA analysis can be performed directly with crude cell extracts avoiding laborious RNA purification. The method was developed for *B. subtilis*, a model organism capable of spore formation and quite abundant in aerosols.

Since spores only contain low amounts of RNA molecules, the idea of this study was to initiate RNA synthesis to increase the detection system sensitivity. At first, the spores are activated at 70°C for 30 min and placed into a medium containing L-alanine as a germination factor. During germination and outgrowth the activated spores synthesize RNA, proteins, and ATP. Chromosomal replication is initiated after 0.5 h (Garrick-Silversmith and Torriani, 1973) and the first cell division takes place after approximately 70 min (Keijser et al., 2007). Depending on the initial amount of spores, cells are disrupted either directly after the germination or after the enrichment cultivation required for the accumulation of necessary amount of RNA molecules. The presented method allows the use of crude cells lysates as sample material for the RNA detection, and no further RNA purification is needed (Rautio et al., 2003; Thieme et al., 2008). We believe that this is a strong advantage for using the sandwich hybridization assay for the RNA detection compared to reverse transcriptase real time PCR.

The assay is based on the hybridization event of the target RNA with two oligonucleotide probes. Alkaline phosphatase attached to one of the probes generates a fluorescent signal used for quantification. The SHA has a potential to be automated, e.g., by the use of an electrical chip reader (Gabig-Ciminska et al., 2004).

After the activation the spores germinated very quickly. The first increase of the OD_600_ was noticed already after 1 h for the cultures with an initial amount of 10^6^ spores per ml. The detectable SHA signal for these cultures was observed after 0.5 h of germination. Since the RNA synthesis in germinating *B. subtilis* spores starts within about 5 min after spore activation (Matsuda and Kameyama, 1986), the cells have accumulated a detectable amount of RNA molecules already after 30 min. Furthermore, at this moment the spore core is rehydrated making cells more accessible to disruption (Sloma and Smith, 1979; Moeller et al., 2006).

The sensitivity of the developed method is not limited due to enrichment cultivation for which the time can be adapted. Very low initial spore amounts in the sample could be detected after about 5 h of enrichment cultivation. Consequently, the maximum duration of the assay for a very low amount of spores including spore activation, enrichment cultivation, and detection with the SHA is about 8 h. The detection limit of the SHA was estimated as 6 × 10^7^ target molecules, which in case of using rRNA as a target corresponds to about 10^3^–10^4^ cells in a sample depending on their growth stage.

By the use of *in vitro* synthesized RNA probes as a standard with the SHA it was possible to estimate the amount of 16S rRNA molecules per cell. As expected, the number of molecules increased during the germination and the outgrowth, reached the maximum at the beginning of the exponential phase of growth, and then slowly decreased. After 0.5 h of germination one cell contained approximately 5.6 (±0.9) × 10^3^ 16S rRNA molecules. Exponentially growing cells contained about 9 × 10^4^ 16S rRNA molecules. These numbers correlate well with the known amount of ribosomes in *Escherichia coli* cells, that is 6700–71,000 depending on the specific growth rate (Bremer and Dennis, 1996).

The 16S rRNA oligonucleotide probes used in this study are capable of detecting most bacteria and are relevant only for method development. Specific probes for the SHA can be designed for almost any organism or groups of organisms if the gene sequence is available. Furthermore, it would be possible to apply the procedure for the analysis of mRNAs and their dynamics during germination. However, due to numerical advantage of ribosomes compared to a specific mRNA species, the method sensitivity would be lower when mRNA is used as a target for the SHA instead of rRNA and additionally the analytical error eventually would be higher due to the low stability of mRNA molecules. For selecting target mRNAs for specific identification of *B. subtilis* a transcription profile of germinated spores (Keijser et al., 2007) and the most abundant proteins of growing *Bacillus* cells have to be reviewed. Protein abundance during the exponential growth of *Bacillus subtilis* has been studied in great detail (Büttner et al., 2001; Eymann et al., 2004). The most abundant proteins in *B. subtilis* cytosolic extracts perform mainly housekeeping functions as components of the translational apparatus (e.g., translation elongation factors, ribosomal proteins), the glycolytic pathways, the tricarboxylic acid cycle, the metabolism of amino and nucleic acids, and protein quality control (e.g., chaperons). The mRNAs of these proteins can be used as a target for the specific detection of *B. subtilis* spores using the presented method.

### Conflict of interest statement

The authors declare that the research was conducted in the absence of any commercial or financial relationships that could be construed as a potential conflict of interest.

## Abbreviations

SHA: sandwich hybridization assay.
